# Case mix, outcome and activity for patients with severe acute kidney injury during the first 24 hours after admission to an adult, general critical care unit: application of predictive models from a secondary analysis of the ICNARC Case Mix Programme Database

**DOI:** 10.1186/cc7003

**Published:** 2008-10-13

**Authors:** Nitin V Kolhe, Paul E Stevens, Alex V Crowe, Graham W Lipkin, David A Harrison

**Affiliations:** 1 Derby City Hospital, Uttoxeter Road, Derby DE22 3NE, UK; 2 Department of Renal Medicine, Kent and Canterbury Hospital, Ethelbert Road, Canterbury, Kent CT1 3NG, UK; 3 Countess of Chester Hospital, Countess of Chester Health Park, Liverpool Road, Chester, Cheshire CH2 1UL, UK; 4 Queen Elizabeth Hospital, Queen Elizabeth Medical Centre, Edgbaston, Birmingham B15 2TH, UK; 5 Intensive Care National Audit & Research Centre (ICNARC), Tavistock House, Tavistock Square, London WC1H 9HR, UK

## Abstract

**Introduction:**

This study pools data from the UK Intensive Care National Audit and Research Center (ICNARC) Case Mix Programme (CMP) to evaluate the case mix, outcome and activity for 17,326 patients with severe acute kidney injury (AKI) occurring during the first 24 hours of admission to intensive care units (ICU).

**Methods:**

Severe AKI admissions (defined as serum creatinine ≥300 μmol/l and/or urea ≥40 mmol/l during the first 24 hours) were extracted from the ICNARC CMP database of 276,326 admissions to UK ICUs from 1995 to 2004. Subgroups of oliguric and nonoliguric AKI were identified by daily urine output. Data on surgical status, survival and length of stay were also collected. Severity of illness scores and mortality prediction models were compared (UK Acute Physiology and Chronic Health Evaluation [APACHE] II, Stuivenberg Hospital Acute Renal Failure [SHARF] T0, SHARF II0 and the Mehta model).

**Results:**

Severe AKI occurred in 17,326 out of 276,731 admissions (6.3%). The source of admission was nonsurgical in 83.7%. Sepsis was present in 47.3% and AKI was nonoliguric in 63.9% of cases. Admission to ICU with severe AKI accounted for 9.3% of all ICU bed-days. Oliguric AKI was associated with longer length of stay for survivors and shorter length of stay for nonsurvivors compared with nonoliguric AKI. Oliguric AKI was associated with significantly greater ICU and hospital mortality (55.8% and 77.3%, respectively) compared with nonoliguric AKI (33.4% and 49.3%, respectively). Surgery during the 1 week before admission or during the first week in the CMP unit was associated with decreased odds of mortality. UK APACHE II and the Mehta scores under-predicted the number of deaths, whereas SHARF T0 and SHARF II0 over-predicted the number of deaths.

**Conclusions:**

Severe AKI accounts for over 9% of all bed-days in adult, general ICUs, representing a considerable drain on resources. Although nonoliguric AKI continues to confer a survival benefit, overall survival from AKI in the ICU and survival to leave hospital remains poor. The use of APACHE II score measured during the first 24 hours of ICU stay performs well as compared with SHARF T0, SHARF II0 and the Mehta score, but it lacks perfect calibration.

## Introduction

Acute kidney injury (AKI) is relatively common in the intensive care setting and has an associated mortality of 50% to 80%, which has remained largely unchanged despite advances in renal replacement therapy (RRT). This has been attributed to the changing pattern of associated pathology and the patient population. AKI is gradually becoming a disease of elderly populations, with the median age increasing from 41 years in the 1950s to 60.5 and 73 years during the periods from 1980 to 1988 and from 1997 to 1998, respectively [1,2]. Patients needing RRT frequently require lengthy intensive care treatment and extensive life support [3,4]. They tend to have multiple co-morbidities, which ultimately influence the outcome. A number of epidemiological studies have been conducted, using various criteria for definition of AKI, that examined various factors that may contribute to mortality [5].

Over the past 25 years, a body of pathophysiological knowledge has been created by intensive care units (ICUs) and that has enabled advances to be made in the treatment of patients. At the same time, a series of tools have been designed to evaluate, from multiple perspectives, the outcomes obtained. Through development of several scoring systems, the intensive care physician can grade the severity of illness in the ICU. The majority of scoring systems focus on mortality as the main outcome measure. Usually, the ability of a particular score to predict mortality is acceptable for a patient group as a whole, but it translates poorly to the individual patient. Heterogeneity of patient populations in ICUs may be a reason for these shortcomings. Several authors have addressed the performance of mortality prediction models in subgroups of patients defined by the same underlying disease or the same cause of intensive care admission. Over the past 2 decades, a variety of illness stratification systems have been employed to analyze the impact that overall co-morbidity has on the outcome of severe AKI, and it is clear that co-morbidity has a major influence on mortality in severe AKI [6-9]. Several mortality prediction models have been developed, both for use in the multidisciplinary ICU as well as for specific use in AKI patients[6,10-16]. Many of these have been developed at a single centre and few were validated outside their original centre.

The Case Mix Programme (CMP) is the national comparative audit of adult, general critical care units in England, Wales and Northern Ireland co-ordinated by the Intensive Care National Audit & Research Centre (ICNARC). The programme collects information about patients during their first 24 hours of admission to the ICU, together with length of stay and mortality outcome data. This study used data from the CMP to describe case mix, outcome and activity for patients with severe AKI during the first 24 hours of admission to ICU.

## Materials and methods

### Case Mix Programme Database

Data were extracted for 276,731 admissions to 170 adult, general critical care units from the CMP Database (CMPD), covering the period from December 1995 to January 2004. Details of the data collection and validation were reported previously [17].

### Selection of cases

The collection of data for the CMP antedated publication and dissemination of the RIFLE (Risk, Injury, Failure, Loss of kidney function, End-stage kidney disease) criteria [18]. Admissions with severe AKI during the first 24 hours after admission to intensive care were therefore identified as those with a highest serum creatinine greater than or equal to 300 μmol/l or a highest serum urea greater than or equal to 40 mmol/l during the first 24 hours after admission to the CMP unit, and not previously requiring chronic RRT. Although the requirement for chronic RRT (defined as either chronic haemodialysis, chronic haemofiltration or chronic peritoneal dialysis) must be documented before admission or at admission to the CMP unit, at the time of this data collection the CMP did not collect data concerning acute RRT. Patients requiring acute RRT during the first 24 hours of admission, who did not achieve the selection criteria, will not have been included in the analysis. Data in the CMP were collected during the first 24 hours of intensive care admission, and therefore do not capture severe AKI developing after the first 24 hours of intensive care admission.

Subgroups of oliguric AKI and nonoliguric AKI were identified by their daily urine output. Oliguria was defined as a daily urine output of less than 400 ml. For admissions staying at least 24 hours in the CMP unit, daily urine output was defined as the urine output during the first 24 hours after admission. For patients admitted and staying at least 8 hours and less than 24 hours in the CMP unit, daily urine output was defined as the urine output for the entire stay divided by the length of stay in the unit as a fraction of 24 hours. Patients admitted and staying less than 8 hours in the CMP unit were excluded from both the oliguric and nonoliguric subgroups because of imprecision in estimating daily urine output in these patients.

### Data

Data were extracted on case mix, outcome and activity as defined below.

#### Case mix

Age at admission and sex were extracted. The following physiological variables, selected *a priori*, were extracted from the first 24 hours in the CMP unit: highest serum creatinine, highest serum urea, lowest serum albumin and lowest haematocrit. Acute severity was summarized using the Acute Physiology and Chronic Health Evaluation (APACHE) II Acute Physiology Score and the APACHE II score. The former encompasses a weighting for acute physiology (defined by derangement from the normal range for 12 physiological variables during the first 24 hours of critical care); the latter additionally encompasses a weighting for age and for a past medical history of specified serious conditions [19].

Surgical status was defined as either nonsurgical, elective surgery or emergency surgery, based on the source of admission to the CMP unit and the National Confidential Enquiry into Patient Outcome and Death classification of surgery, as has previously been described [17].

#### Outcome

Survival data were extracted at discharge from the CMP unit and at ultimate discharge from hospital.

#### Activity

Length of stay in the CMP unit was calculated from the dates and times of admission and discharge. Length of stay in hospital was calculated from the dates of original admission and ultimate discharge.

Transfers in from another critical care unit were identified as admissions whose source of admission to the CMP unit was any critical care unit in the same or another hospital. Readmissions to the unit within the same hospital stay were identified from the postcode, date of birth and sex, and confirmed by the participating units.

### Analyses

#### Descriptive statistics

Variables reflecting case mix, outcome and activity, as defined above, were summarized for all admissions with severe AKI during the first 24 hours in the CMP unit, and for the subgroups of oliguric and nonoliguric AKI.

#### Modelling ultimate hospital mortality

The effect of case mix factors on ultimate hospital mortality was assessed using a multiple logistic regression model. The variables entered into the model were age, sex, APACHE II chronic health conditions (excluding chronic RRT), cardiopulmonary resuscitation (CPR) within 24 hours before admission to the CMP unit, mechanical ventilation during the first 24 hours after admission to the CMP unit, surgery up to 1 week before admission to the CMP unit or within 1 week after admission to and before discharge from the CMP unit, sepsis during the first 24 hours after admission to the CMP unit, oliguria, length of hospital stay before admission to the CMP unit, Glasgow Coma Scale score, and all of the physiological variables from the APACHE II model (except serum creatinine) plus serum albumin.

**Figure 1:**
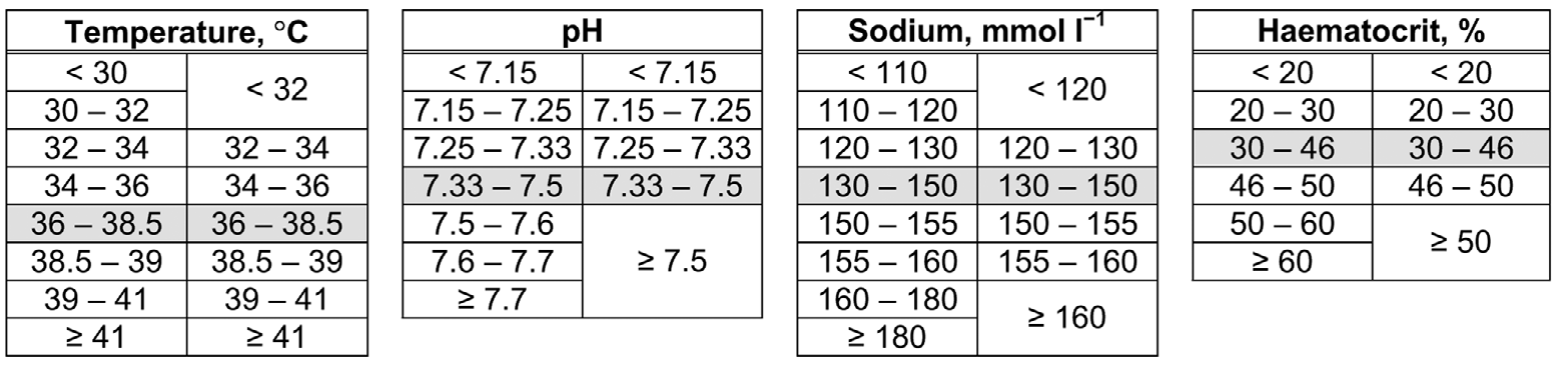
Recoding of APACHE II/III categories for logistic regression model. Recoding performed to eliminate categories containing fewer than 50 admissions in the multiple logistic regression model. Grey cells denote reference categories. APACHE, Acute Physiology and Chronic Health Evaluation.

Sepsis was classified according to the definition proposed by the American College of Chest Physicians/Society of Critical Care Medicine Consensus Conference [20] as evidence of infection plus at least two systemic inflammatory response syndrome criteria. These definitions were matched as closely as possible using data available in the CMPD, as has been described previously [21]. Length of hospital stay before admission to the CMP unit was identified from the date of admission to the CMP unit and the date of original admission to hospital, and was categorized as 0 days (hospital and critical care unit admission occurred on the same calendar day), 1 day, 2 to 6 days, or 7 or more days. Age and Glasgow Coma Scale score were modelled as having a linear effect on the log odds. All other variables were modelled categorically, using the categories from APACHE II or APACHE III as appropriate for the physiological variables but fitting new weights to each category [6,19]. When a variable was present in both APACHE II and III, the categorization giving the greatest number of categories was selected. Categories from APACHE II were used to model temperature, mean arterial pressure, arterial pH, serum sodium, serum potassium, haematocrit and white blood cell count. Categories from APACHE III were used to model heart rate, respiratory rate, oxygenation (either alveolar-arterial oxygen gradient or arterial oxygen tension, depending on the inspired oxygen level) and serum albumin. Before modelling, adjacent categories were collapsed to ensure that all categories contained at least 50 admissions (Figure 1). Admissions missing any routinely measured physiological variables (temperature, blood pressure, heart rate or respiratory rate) were excluded from the modelling. All other missing physiological values were assumed normal and were allocated to the category corresponding to zero APACHE II/III points.

#### Comparison of existing mortality prediction models

Data from the CMPD were used to approximate, as closely as possible, three existing published mortality prediction models for AKI: the two Stuivenberg Hospital Acute Renal Failure (SHARF) scores, and the model of Mehta and coworkers [22-24].

The SHARF score should be evaluated at two specific time points: T0, at the time of diagnosis of AKI (or the time of admission to the unit if AKI had been diagnosed before admission); and T48, 48 hours later. The scores consist of weights for age, albumin and prothrombin time at T0, and indicators of mechanical ventilation and heart failure at the time of scoring (T0 or T48). The physiology data in the CMPD are collected as worst values over the first 24 hours after admission to the CMP unit, and we took these to represent measurements at T0 for albumin and prothrombin time, with the recording of a ventilated respiratory rate during the first 24 hours after admission taken to represent ventilation at T0. The physiological variables used to define heart failure in SHARF are not recorded in the CMP, and so we relied on the use of vasoactive drugs or the clinical recording of heart failure as a primary or secondary reason for admission to intensive care or as a condition relevant to the admission as a proxy for this variable. No information on mechanical ventilation or heart failure at 48 hours can be established from CMP data, so we did not attempt to construct the T48 score. The SHARF score takes the form of an integer score, with higher scores indicating worse prognosis. A logistic model is used to convert the score to a probability of hospital mortality. The coefficients for this model were not reported, but from a graphical representation of the conversion we estimate the mortality equation at T0 to be as follows: log odds of hospital mortality = 0.0624 × (SHARF T0) – 8.88.

SHARF II is a revision of SHARF. The SHARF II0 score includes bilirubin, sepsis and hypotension at T0, in addition to all of the other parameters in SHARF T0. The same limitations in the definitions as for SHARF T0 also apply to SHARF II0. The mortality equation for SHARF II0 was estimated to be as follows: log odds of hospital mortality = 0.0556 × (SHARF II0) – 2.99.

The Mehta model consists of weights for age, sex, blood urea nitrogen, creatinine, haematological failure, liver failure, respiratory failure, heart rate and urine output. The patients included in the development of the Mehta model were all patients receiving a nephrology consultation for AKI while in the unit or those transferred to the unit with AKI. Measurements were taken on the day of the nephrology consultation, or on the first day in the unit for those transferred in with AKI. The worst values measured over the first 24 hours in the CMP unit were used for blood urea nitrogen, creatinine and heart rate. Urine output was taken to be the total urine output during the first 24 hours in the CMP unit. The physiological definitions of haematological failure and respiratory failure were matched very closely, with the exceptions that a requirement for platelet transfusions as an indicator of haematological failure, and ventilator dependence as an indicator of respiratory failure could not be identified in the CMPD. The definition of liver failure in the Mehta model is, 'Acute liver failure with elevations in bilirubin levels (total and direct), AST (asparate aminotransferase) and ALT (alanine aminotransferase), and alkaline phosphatase greater than two times normal, increase in prothrombin time and INR (international normalized ratio) of > 1.5 (for patients with pre-existing chronic liver disease, documented evidence of worsened liver function, and presence of encephalopathy.' The only variables from this definition that were available in the CMPD were total serum bilirubin and prothrombin time, and no specific cut-offs are identified in the Mehta model to indicate acute liver failure. We elected to define acute liver failure as a highest total serum bilirubin above 100 μmol/l.

For comparison, we also evaluated the mortality probability from the UK APACHE II model, representing a generic model for critical care admissions. The models were assessed for discrimination using the area under the receiver operating characteristic (ROC) curve, for calibration by the mortality ratio (observed over expected deaths), the Hosmer-Lemeshow C-statistic and Cox's calibration regression; and for overall fit using the R statistic from Shapiro's Q, representing the geometric mean of the probability assigned to the true outcome [25-28]. The models were assessed in the subset of admissions for which all three models could be calculated.

All analyses were performed using Stata 8.2 (Stata Corp LP, College Station, Texas, USA).

## Results

### Data

Of 276,731 admissions to 170 adult critical care units in the CMPD, 17,326 (6.3%) were identified as having severe AKI during the first 24 hours in the unit. Table 1 describes measures of case mix, outcome and activity for these admissions and for the oliguric and nonoliguric AKI subgroups.

**Table 1:** Case mix, outcome and activity for patients with acute kidney injury during the first 24 hours following admission to intensive care

Category	Parameter	All patients (n = 17,326)	Oliguric^a ^(n = 5,687)	Nonoliguric^a ^(n = 10,133)
Case mix	Age (years; mean ± SD)	63.2 ± 15.6	63.5 ± 15.0	63.1 ± 15.7
	Sex (male; n [%])	11,511 (66.4)	3,617 (63.6)	6,925 (68.3)
	Surgical status (n [%])			
	Nonsurgical	14,479 (83.7)	5,005 (88.1)	8,125 (80.3)
	Elective surgery	961 (5.6)	187 (3.3)	717 (7.1)
	Emergency surgery	1,868 (10.8)	487 (8.6)	1,280 (12.6)
	Serum parameters (mean ± SD)			
	Highest serum creatinine (μmol/l)	459 ± 232	502 ± 244	431 ± 216
	Highest serum urea (mmol/l)	32.2 ± 27.3	29.5 ± 15.2	33.9 ± 32.3
	Lowest serum albumin (g/l)	21.3 ± 7.2	20.5 ± 6.9	21.6 ± 7.2
	Lowest haematocrit (%)	28.6 ± 6.4	28.1 ± 6.3	28.8 ± 6.3
	APACHE II (mean ± SD)			
	APS^b^	20.2 ± 7.0	24.6 ± 6.5	17.9 ± 6.1
	Total score^b^	24.8 ± 7.5	29.3 ± 7.0	22.4 ± 6.5

Outcome	Mortality (n [%])			
	CMP unit	7,508 (43.3)	3,176 (55.8)	3,387 (33.4)
	Any hospital^c^	9,725 (58.6)	3,850 (70.3)	4,770 (49.3)

Activity	Length of stay (days; median [IQR])			
	CMP unit: survivors	4.1 (1.7–10.3)	5.7 (2.1–13.2)	4.0 (1.8–9.8)
	CMP unit: nonsurvivors	2.0 (0.8–6.1)	1.7 (0.9–5.0)	3.4 (1.5–8.9)
	Any hospital^c^: survivors	31 (17–54)	38 (24–62)	29 (16–51)
	Any hospital^c^: nonsurvivors	8 (3–20)	7 (2–18)	11 (4–23)
	Transfers from another ICU (n [%])	1,355 (7.8)	561 (9.9)	730 (7.2)
	Readmissions within hospital stay (n [%])	388 (2.2)	116 (2.0)	221 (2.2)

^a^Oliguria is defined as urine output < 400 ml/24 hours; admitted patients staying less than 8 hours in the ICU excluded. ^b^APACHE II exclusions: age < 16 years; ICU stay < 8 hours; readmissions within same hospital stay; transfers from another ICU; admissions following coronary artery bypass surgery; and admissions for primary burns. ^c^Excluding readmissions within the same hospital stay. APACHE, Acute Physiology and Chronic Health Evaluation; APS, Acute Physiology Score; CMP, Case Mix Programme; ICU, intensive care unit; IQR, interquartile range; SD, standard deviation.

### Case mix

The mean age of all patients admitted with severe AKI was 63.2 years. There was no age difference between patients with oliguric and nonoliguric AKI. Sixty-six per cent of admitted patients with AKI were male. The mean APACHE II score was 24.8, as compared with 16.5 for all admissions included in the CMPD [17]. Oliguria was associated with higher APACHE II score and Acute Physiology Score. Eighty-four per cent of all admissions were nonsurgical, and only 16% were surgical (emergency surgery/elective surgery ratio 1.94/1).

### Outcome

Overall mortality in the CMP unit was 43.3%, rising to 58.6% at ultimate hospital discharge. Mortality was higher for admissions with oliguric AKI (55.8% at unit discharge and 70.3% at hospital discharge) than for admissions with nonoliguric AKI (33.4% and 49.3%).

### Activity

The median length of stay in the CMP unit was 4.1 days for survivors and 2.0 days for nonsurvivors. These compare with median values of 1.7 days and 2.0 days, respectively, for all admissions included in the CMPD. Patients admitted with severe AKI during the first 24 hours of intensive care accounted for 9.3% of all intensive care bed-days in these units. Median hospital length of stay was 31 days for survivors and 8 days for nonsurvivors, as compared with 16 days for survivors and 9 days for nonsurvivors in the CMPD as a whole. Oliguria was associated with longer lengths of stay for survivors and shorter lengths of stay for nonsurvivors. Eight per cent of all patients admitted were transferred in from other critical care units, and 2% were readmitted from within the same hospital stay. Patients admitted with oliguric AKI were more likely to be transferred in from another critical care unit than those with nonoliguric AKI.

### Relationship of case mix factors with ultimate hospital mortality

Table 2 shows the results of the multiple logistic regression model. All factors included in the model were significantly associated with hospital mortality. The following factors were associated with increased odds of mortality: increasing age, male sex, presence of past medical history conditions, CPR during the 24 hours before admission to the CMP unit, mechanical ventilation during the first 24 hours after admission to the CMP unit, oliguria, hospital stay of at least 1 week before admission to the CMP unit, extreme (low or high) temperature, low mean arterial pressure, extreme heart rate, extreme respiratory rate, high respiratory rate, high alveolar-arterial oxygen gradient, low pH, abnormal serum sodium, high serum potassium, low serum albumin, high haematocrit, low white blood count, and low Glasgow Coma Scale score. Surgery during the week before admission or during the first week in the CMP unit was associated with decreased odds of mortality. The presence of sepsis was also associated with, and was a significant predictor of, higher hospital mortality (61% versus 53%).

**Table 2:** Effects of case mix factors on ultimate hospital outcome in admissions with acute kidney injury

Case mix factor		Deaths (n)	n	%	Adjusted OR (95% CI)	*P *value
Age (years)	< 45	817	2,128	38.4	1.48 (1.44–1.52) per 10 year increase	< 0.001
	45–54	846	1,776	47.6		
	55–64	1,671	3,086	54.1		
	65–74	2,991	4,866	61.5		
	75+	2,553	3,720	68.6		

Sex	Female	2,905	5,217	55.7	Reference	0.004
	Male	5,973	10,359	57.7	1.13 (1.04–1.22)	

Past medical history	Absent	7,355	13,298	55.3	Reference	< 0.001
	Present	1,523	2,278	66.9	1.82 (1.63–2.03)	

CPR before admission	No	7,679	14,062	54.6	Reference	< 0.001
	Yes	1,199	1,514	79.2	1.64 (1.41–1.90)	

Mechanical ventilation	No	2,086	5,250	39.7	Reference	< 0.001
	Yes	6,792	10,326	65.8	1.93 (1.76–2.12)	

Surgery within 1 week of admission	No	6,324	10,751	58.8	Reference	< 0.001
	Yes	2,554	4,825	52.9	0.80 (0.73–0.87)	

Sepsis	No	4,362	8,207	53.1	Reference	< 0.001
	Yes	4,516	7,369	61.3	1.14 (1.06–1.21)	

Oliguria	No	4,766	9,660	49.3	Reference	< 0.001
	Yes	3,844	5,466	70.3	1.90 (1.75–2.07)	

Length of stay before unit admission	0 days	2,451	4,261	57.5	Reference	< 0.001
	1 day	1,952	3,666	53.2	0.90 (0.81–1.01)	
	2–6 days	2,269	4,133	54.9	1.00 (0.90–1.12)	
	7+ days	2,199	3,508	62.7	1.54 (1.38–1.73)	

Temperature (°C)^a^	< 32	68	89	76.4	1.96 (1.12–3.40)	< 0.001
	32–34	399	557	71.6	1.32 (1.05–1.65)	
	34–36	2,779	4,595	60.5	1.11 (1.01–1.22)	
	36–38.5	2,918	5,789	50.4	Reference	
	38.5–39	882	1,680	52.5	0.96 (0.85–1.10)	
	39–41	1,614	2,595	62.2	1.16 (1.03–1.30)	
	≥ 41	185	217	85.3	3.35 (2.18–5.15)	

Mean arterial pressure (mmHg)^a^	< 50	2,923	3,803	76.9	2.08 (1.80–2.41)	< 0.001
	50–70	4,164	7,013	59.4	1.45 (1.28–1.65)	
	70–110	634	1,682	37.7	Reference	
	110–130	724	1,988	36.4	0.89 (0.77–1.04)	
	130–160	366	955	38.3	0.94 (0.78–1.13)	
	≥ 160	48	96	50.0	1.33 (0.83–2.12)	

Heart rate (beats/minute)b	< 40	134	187	71.7	1.42 (0.97–2.08)	< 0.001
	40–50	179	324	55.2	1.14 (0.87–1.50)	
	50–100	1,229	2,775	44.3	Reference	
	100–110	1,044	2,085	50.1	1.18 (1.03–1.35)	
	110–120	1,287	2,408	53.4	1.26 (1.11–1.44)	
	120–140	2,543	4,251	59.8	1.46 (1.30–1.64)	
	140–155	1,262	1,878	67.2	1.66 (1.43–1.93)	
	≥ 155	1,173	1,623	72.3	1.94 (1.66–2.28)	

Respiratory rate (breaths/minute)^b^	< 6	403	628	64.2	1.05 (0.85–1.30)	0.001
	6–12	1,836	3,509	52.3	1.00 (0.88–1.13)	
	12–14	1,771	2,867	61.8	1.19 (1.05–1.36)	
	14–25	1,395	2,397	58.2	Reference	
	25–35	2,007	3,784	53.0	1.04 (0.92–1.18)	
	35–40	689	1,109	62.1	1.33 (1.12–1.58)	
	40–50	608	1,015	59.9	1.16 (0.97–1.39)	
	≥ 50	135	203	66.5	1.49 (1.05–2.13)	

Oxygenation (mmHg; A-aDO_2 _[FiO_2 _≥0.5])^b^	A-aDO_2 _(FIO_2 _≥ 0.5)					< 0.001
	< 250	1,238	2,338	53.0	Reference	
	250–350	1,576	2,540	62.0	1.25 (1.12–1.40)	
	350–500	1,034	1,453	71.2	1.68 (1.45–1.94)	
	≥ 500	1,480	1,951	75.9	1.94 (1.69–2.23)	
	PaO_2 _(FIO_2 _< 0.5)					
	< 50	114	206	55.3	1.10 (0.80–1.53)	
	50–70	918	1,786	51.4	1.11 (0.98–1.26)	
	70–80	786	1,637	48.0	1.02 (0.90–1.16)	
	≥ 80	1,732	3,665	47.3	Reference	

Arterial pH^a^	< 7.15	1,380	1,676	82.3	2.13 (1.82–2.49)	< 0.001
	7.15–7.25	1,871	2,655	70.5	1.63 (1.46–1.83)	
	7.25–7.33	2,177	3,744	58.1	1.20 (1.09–1.32)	
	7.33–7.5	3,290	7,156	46.0	Reference	
	≥ 7.5	160	345	46.4	0.98 (0.76–1.26)	

Serum sodium (mmol/l)^a^	< 120	187	326	57.4	1.49 (1.14–1.94)	< 0.001
	120–130	1,506	2,654	56.7	1.19 (1.07–1.32)	
	130–150	6,590	11,687	56.4	Reference	
	150–155	358	537	66.7	1.45 (1.17–1.80)	
	155–160	153	221	69.2	1.48 (1.06–2.07)	
	≥ 160	84	151	55.6	0.92 (0.63–1.36)	

Serum potassium (mmol/l)^a^	< 2.5	56	110	50.9	0.67 (0.43–1.05)	< 0.001
	2.5–3	302	535	56.4	0.93 (0.75–1.14)	
	3–3.5	1,106	2,008	55.1	0.91 (0.81–1.02)	
	3.5–5.5	4,639	8,297	55.9	Reference	
	5.5–6	1,137	1,858	61.2	1.17 (1.04–1.32)	
	6–7	1,244	2,028	61.3	1.13 (1.00–1.27)	
	≥ 7	394	740	53.2	0.79 (0.66–0.95)	

Serum albumin (g/l)^b^	< 20	3,544	5,499	64.4	1.21 (1.11–1.33)	< 0.001
	20–25	1,834	3,356	54.6	1.02 (0.93–1.13)	
	25–45	3,469	6,635	52.3	Reference	
	≥ 45	31	86	36.0	0.55 (0.33–0.93)	

Haematocrit (%)^a^	< 20	612	1,008	60.7	0.99 (0.84–1.17)	0.011
	20–30	4,748	8,536	55.6	0.89 (0.82–0.97)	
	30–46	3,168	5,473	57.9	Reference	
	46–50	218	335	65.1	1.28 (0.98–1.68)	
	≥ 50	132	224	58.9	0.99 (0.71–1.38)	

White blood count (×1,000/mm^3^)^a^	< 1	176	223	78.9	2.52 (1.73–3.67)	< 0.001
	1–3	506	680	74.4	1.77 (1.44–2.18)	
	3–15	3,862	7,468	51.7	Reference	
	15–20	1,756	3,071	57.2	1.11 (1.00–1.23)	
	20–40	2,278	3,681	61.9	1.21 (1.09–1.33)	
	≥ 40	300	453	66.2	1.16 (0.91–1.47)	

Glasgow Coma Scale scored	3–4	1,596	2,019	79.0	1.07 (1.06–1.08) per decrease of 1 point	< 0.001
	5–12	1,178	1,924	61.2		
	13–14	1,099	1,880	58.5		
	15	5,005	9,753	51.3		

All of the factors emerged as statistically significant (*P *< 0.005) in the multiple logistic regression analysis. Exclusions were as follows: readmissions within the same hospital stay; admissions with length of ICU stay < 8 hours; admissions of patients aged < 16 years; admissions missing age, sex, surgical status, temperature, mean arterial pressure, heart rate or respiratory rate. Admitted patients with missing values for any other physiology variables are assumed to be normal and are placed in the reference category. ^a^Categories from APACHE II (reference category equivalent to zero APACHE II points) ^b^Categories from APACHE III (reference category equivalent to zero APACHE III points). ^c^Pre-sedation values used for admissions sedated or paralyzed during the first 24 hours in ICU. A-aDO_2_, alveolar-arterial oxygen gradient; APACHE, Acute Physiology and Chronic Health Evaluation; CI, confidence interval; CPR, cardiopulmonary resuscitation; FiO_2_, fractional inspired oxygen; ICU, intensive care unit; OR, odds ratio.

### Comparison of existing mortality prediction models

**Figure 2:**
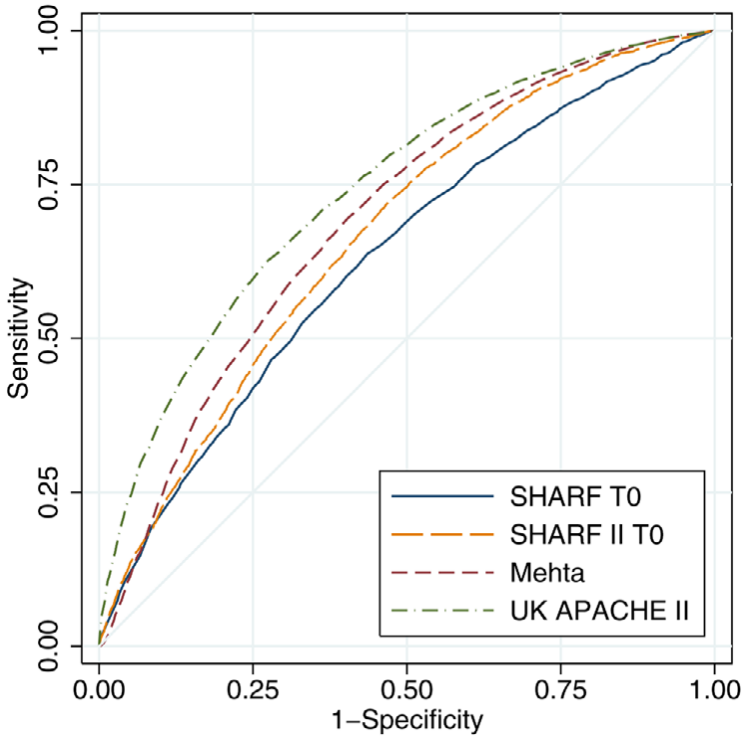
ROC curves for SHARF T0, SHARF II0, Mehta and UK APACHE II. APACHE, Acute Physiology and Chronic Health Evaluation; SHARF, Stuivenberg Hospital Acute Renal Failure.

**Figure 3:**
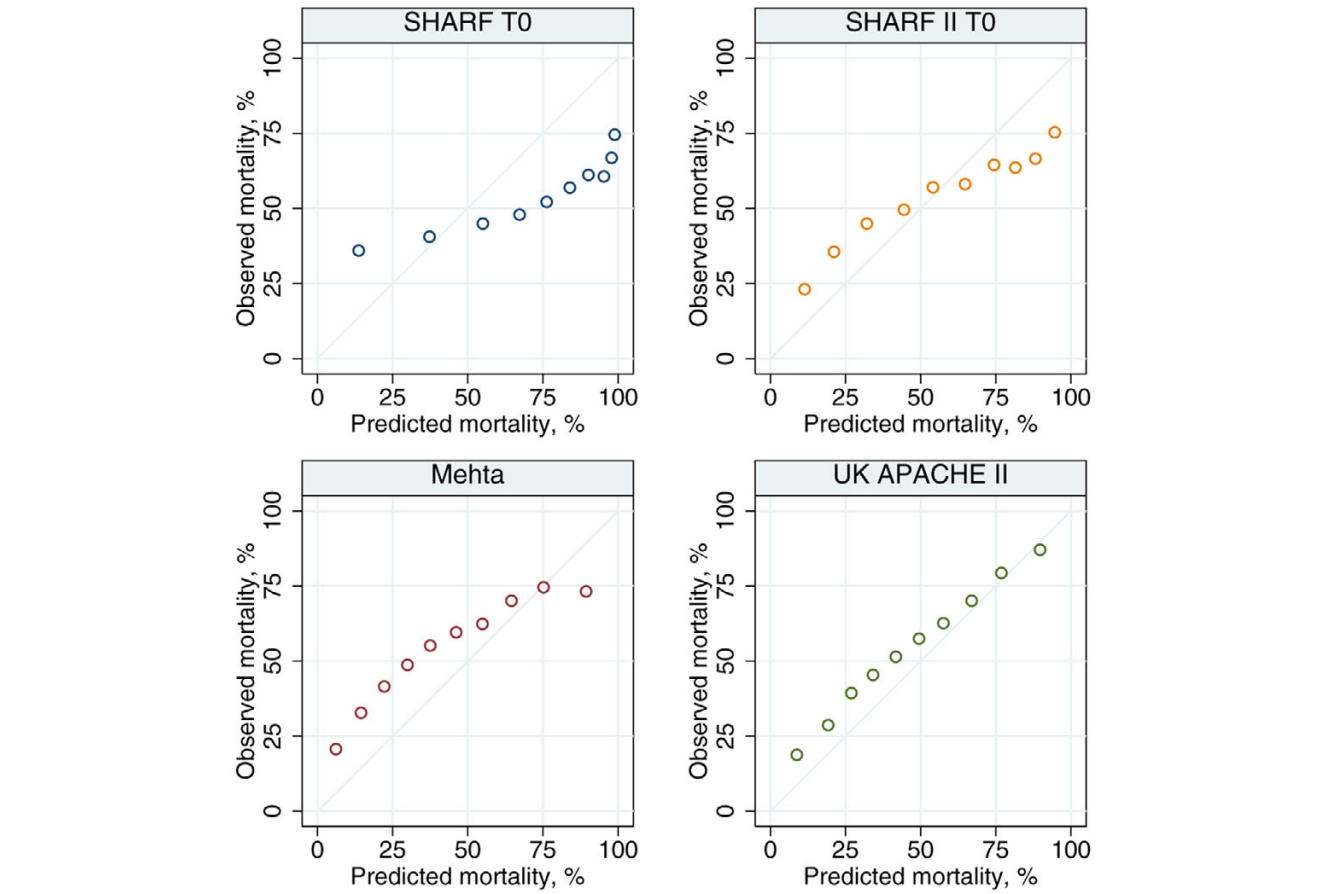
Calibration plots for SHARF T0, SHARF II0, Mehta and UK APACHE II. Observed mortality plotted against deciles of predicted mortality. Diagonal line indicates perfect calibration. APACHE, Acute Physiology and Chronic Health Evaluation; SHARF, Stuivenberg Hospital Acute Renal Failure.

Of the 17,326 patients admitted with severe AKI identified during the first 24 hours in intensive care, 14,118 (81.5%) had sufficient data to calculate the SHARF scores, Mehta probability and UK APACHE II probability; met the inclusion criteria for all three models; and had data on mortality at hospital discharge. Measures of discrimination, calibration and overall accuracy for these models are given in Table 3. Figure 2 shows the ROC curves for the three models, and Figure 3 shows calibration plots of observed against predicted mortality.

**Table 3:** Measures of discrimination, calibration and model fit for SHARF T0, Mehta and UK APACHE II

	SHARF T0	SHARF II0	Mehta	UK APACHE II
Discrimination (AUC [95% CI])	0.632 (0.624–0.640)	0.668 (0.660–0.676)	0.693 (0.686–0.701)	0.74 (0.733–0.748)

Calibration (mortality ratio [95% CI])	0.759 (0.747–0.771)	0.950 (0.936–0.965)	1.223 (1.205–1.242)	1.147 (1.129–1.165)
Hosmer-Lemeshow (C-statistic [*P *value])	20,894 (< 0.001)	2,575 (< 0.001)	2,170 (< 0.001)	575 (< 0.001)
Cox's regression calibration:				
Slope (95% CI)	0.247 (0.229–0.266)	0.415 (0.390–0.439)	0.444 (0.417–0.470)	0.753 (0.720–0.787)
Intercept (95% CI)	-0.195 (-0.243 to -0.153)	0.005 (-0.031 to -0.040)	0.314 (0.278–0.350)	0.306 (0.269–0.343)
χ^2 ^_(1) _(*P *value)^a^	11,598 (< 0.001)	2,303 (< 0.001)	2,602 (< 0.001)	596 (< 0.001)

Fit (Shapiro's Q [R statistic])	0.392	0.491	0.487	0.539

^a^Chi-squared statistic on one degree of freedom (and associated *P *value) from a likelihood ratio test of slope = 1, intercept = 0 in Cox's regression calibration model. APACHE, Acute Physiology and Chronic Health Evaluation; AUC, area under the receiver operating characteristic curve; CI, confidence interval; SHARF, Stuivenberg Hospital Acute Renal Failure.

The UK APACHE II model exhibited the best discrimination and the best calibration, although the null hypothesis of perfect calibration was strongly rejected (*P *< 0.001) by both the Hosmer-Lemeshow test and Cox's calibration regression. Overall, UK APACHE II and Mehta under-predicted the number of deaths (mortality ratio > 1), whereas SHARF T0 and SHARF II0 over-predicted the number of deaths (mortality ratio < 1). SHARF T0, SHARF II0 and Mehta exhibited very poor overall fit by Shapiro's Q, with a value of R < 0.5 indicating a worse fit than would be obtained by predicting a constant mortality probability of 0.5.

## Discussion

In this study we describe the demographics, characteristics, length of stay and outcome of a nationwide cohort of patients with both dialysis dependent and nondependent severe AKI in the ICU – the largest such cohort ever described. In contrast to other studies, in which a major limitation has been lack of heterogeneity in the study population, the CMP is a national comparative audit of adult general critical care units in England, Wales and Northern Ireland.

Our study has some important limitations. The definition of AKI we used was that recommended at the time by the UK Renal Association standards document [18] and roughly corresponds to the 'failure' category in the RIFLE criteria or to stage 3 of the Acute Kidney Injury Network criteria. Caution therefore must be exercised in extrapolating our results to milder forms of AKI. Although the CMP captured patients known to be receiving RRT for chronic irreversible kidney disease at the time of data collection, it did not capture data concerning provision of acute RRT during the first 24 hours in ICU, and we were therefore unable to perform subgroup analysis for patients receiving RRT. The CMP does not capture pre-ICU admission baseline serum creatinine values, and it is conceivable that a few patients might have had very advanced chronic kidney disease but were not yet receiving chronic RRT, as opposed to severe AKI. As with any large database, the CMPD is not immune to missing data. In the CMPD, 2% of the physiological data were missing and this was assumed normal, but the fact that ICU patients who generate missing values in observational studies are more likely to less severely ill and have better outcomes is well established [19]. Finally, in evaluating the SHARF scores we used the use of vasoactive drugs as a surrogate for heart failure, and it is possible that patients receiving noradrenaline for severe sepsis were misclassified as having heart failure. This may weaken our conclusions regarding the validity of the SHARF scores.

We found the prevalence of severe AKI to be 6.3% of all ICU admissions. This is keeping with the prevalence of 5.73% reported by Lins and coworkers [22]. Other studies have found a similar prevalence of severe AKI in ICU using a definition similar to the one that we used [29-31].

Part of the basis for the RIFLE classification of AKI was that even mild impairment in renal function has an impact on mortality. Unsurprisingly, studies using the RIFLE criteria therefore report a higher overall prevalence of AKI. Hoste and coworkers [31] recently reported an overall prevalence of AKI of 67.2%; limiting cases to those with RIFLE class F reduced this to 28%. Others have reported lower figures. Ostermann and colleagues [5] found the overall incidence of AKI to be 35.8%, whereas Uchino and coworkers [32] found the incidence of AKI to be 14.7%. AKI is a dynamic process, and patients may progress from 'risk' to 'injury' to 'failure'. Although Ostermann and colleagues had more patients in the 'risk' class, Hoste and coworkers found that more than 50% of their 'risk' patients had progressed to 'injury' or to 'failure'. The variability in the incidence of AKI, even using the same definition of AKI, can be partly attributed to the different case mix of patients in ICU in Northern America, Australia and the UK.

The mean age of admitted patients in the present study was 63.2 years, and this is similar to that identified in many other studies of AKI in the ICU, indicating that AKI is gradually becoming a disease of the elderly population [5,9,33,34] Although Mehta and coworkers [23] reported a surgical predominance of causes of AKI (63.1%), the aetiology of AKI in the adult, general ICU has become predominantly nonsurgical and ranges from 65% to 85% of all admissions [29,35].

In our study, the ICU mortality of severe AKI was 43.3%, increasing to 58.6% at the time of hospital discharge. It is important to appreciate that mortality in all classes of AKI is increased even after discharge from ICU, and hospital mortality is therefore a more accurate reflection of the outcome from AKI in the ICU. In another UK study, Abosaif and coworkers [36] applied the RIFLE classification retrospectively to their ICU patients and found an ICU mortality of 74.5% in the 'failure' class of AKI, whereas their overall ICU mortality rate was 47.5%. Hoste and colleagues [31] reported a gradual increase in mortality in different classes of AKI, with an ICU mortality of 26.3% in patients with the 'failure' class of AKI. Åhlström and coworkers [37] found a mortality of 23% in the 'failure' class of AKI. A recent study [30] investigated the epidemiology of all AKI in the ICU in 54 study centres, irrespective of RRT requirement. The authors reported an ICU mortality of 52% and a hospital mortality of 60.3%.

AKI patients tend to be sicker, have multiple co-morbidities and require longer ICU stay compared with patients without AKI. In the present study, the presence of severe AKI increased both the median length of ICU and hospital stay. The presence of oliguria further prolonged the length of ICU and hospital stay.

The presence of oliguria has long been considered a negative predictor of patient survival and a factor associated with delayed renal recovery [7,13,38,39]. However, strategies aiming to convert oliguric states to nonoliguric states have not been successful in terms of improving outcomes [40,41]. In the present study the median length of ICU stay for oliguric AKI survivors was increased in comparison with that in nonoliguric AKI survivors. The outcome was also significantly poorer, with both ICU and hospital mortality considerably worse for oliguric AKI than for nonoliguric AKI. Oliguric patients generally tend to have more advanced AKI, and in our study we found these patients to have a higher APACHE II score (29.3 versus 22.4).

We investigated the effects of case mix factors on ultimate hospital outcome in this large cohort of patients with severe AKI. Increasing age was found to be a negative predictor of survival. Several studies have also identified age as an adverse prognostic factor in AKI, whereas others have not found this association [5,13,16,19,32,42]. With increasing age the number of co-morbidities increases, and this can have deleterious effects on survival. Increasing length of stay before admission to ICU (> 7 days) was associated with increased risk for death in the present study. Brivet and coworkers [35] found a hospital mortality of 50% in patients admitted directly to ICU, in contrast to 66% for patients transferred to ICU from within the hospital. Multiple and increasing organ dysfunction before ICU transfer was the probable cause. Studies have shown that 'isolated AKI' occurring as a result of contrast nephropathy due to routine radiological procedures has a much lower mortality [8,9]. Proven or suspected sepsis was predictive of death in several studies [7,30,35,43] and this too was the case in our study. As with previous studies, hypotension and need for mechanical ventilation were also predictors of death in our study [5,13,16,39,44,45].

Many studies have attempted to find the best possible severity of illness scoring system, but each scoring system developed has been limited by the lack of heterogeneity in the population studied and/or development of the scoring system based on findings from a single centre study [23,36,37,39,46,47]. In addition, severity of illness scores have been applied at different time frames within the ICU stay, for example at admission to ICU, within 24 hours of admission to ICU and at the initiation of RRT. A particular goal for any of the severity of illness scores in AKI should be to identify patients for whom RRT will be futile because of 100% mortality despite dialysis. In this large study, we estimated the conventional severity of illness risk score commonly used in the UK (APACHE II) and compared the area under the ROC curve with that for the SHARF T0, SHARF II0 and Mehta severity of illness scores. We found the area under the ROC curve to be best for APACHE II (0.740), but it over-predicted death (mortality ratio > 1). The area under the ROC curve for the Mehta, SHARF T0 and SHARF II0 scores was 0.693, 0.632 and 0.668, respectively, and all of them under-predicted death, although SHARF II0 performed better than SHARF T0. Douma and coworkers [46] compared 11 different mortality prediction models in ICU patients with dialysis-dependent AKI and reported good performance of the APACHE III and Liano models (area under the ROC curve of 0.74 and 0.78, respectively). In contrast, Lins and coworkers [22] found the ROC values of the Liano model and APACHE II to be lower than that of the SHARF T0 (0.82) scores. The SHARF score, developed in a single-centre study, was found to be less precise when tested in new and different multicentre ICU cohorts (area under the ROC curve values of 0.67 and 0.78 at 0 and 48 hours in multicentre cohorts, respectively, versus 0.87 and 0.9 in the original single centre cohort). Lins and coworkers [24] reanalyzed the multicentre data after adding three more variables in the modified SHARF II score and demonstrated improved discrimination (area under the ROC curve values of 0.82 and 0.83 at 0 and 48 hours, respectively).

Abosaif and coworkers [36] found that the Simplified Acute Physiology Score II and the APACHE II score were more reliable in patients with either the 'risk' or 'injury' RIFLE classes than for the 'failure' class. Åhlström and coworkers [37] found that the Sequential Organ Failure Assessment score (area under the AUC curve 0.725) performed better than APACHE II score (area under the AUC curve 0.703). Chertow and coworkers [39] compared their PICARD (Program to Improve Care in Acute Renal Disease) model with eight generic and three AKI-specific scores at three different time points: AKI diagnosis, the day of consultation and the day of first procedure. Among the generic models, they found that the Sequential Organ Failure Assessment score performed the best when applied at the two later points. The Cleveland Clinic Foundation score performed best among patients requiring RRT

So what can we conclude then about severity of illness scores in ICU patients with AKI? To date no AKI-specific severity of illness scoring method has exhibited excellent predictive power for mortality. There could be several reasons for this. AKI is a dynamic process, with increasing mortality associated with increasing severity of AKI. At present there is no consensus regarding the timing of application of mortality prediction scores. The various mortality prediction scores have been developed using different definitions of AKI.

In the present study the UK APACHE II model exhibited the best discrimination and the best calibration, although the null hypothesis of perfect calibration was strongly rejected. The Mehta score under-predicted mortality (ratio > 1). Although possibly influenced by an inaccurate definition of heart failure, the SHARF T0 and SHARF II0 over-predicted mortality (ratio < 1). However, even this caveat could not possibly account for the large deficit in discrimination, perhaps serving to illustrate that specific models derived in small groups of patients are unlikely to outperform general models derived in large cohorts.

## Conclusion

The results of the present study confirm that severe AKI in ICU patients presents an excess risk for ICU and in-hospital mortality, and doubles ICU and hospital lengths of stay, irrespective of the requirement for RRT. Patients with severe AKI in the ICU accounted for 9.3% of all ICU bed-days, and these figures provide a basis for an estimation of the minimum service required to provide ICU services for the treatment of severe AKI and the potential associated costs. Patients with oliguric AKI had even worse mortality, and those surviving had even longer lengths of ICU and hospital stay.

The aetiology of ICU severe AKI in the UK is predominantly nonsurgical, and the mean age of ICU AKI in the UK is increasing. Increasing age; past medical history; CPR; mechanical ventilation; sepsis; oliguria; length of stay more than 7 days before ICU admission; extremes of mean arterial pressure, heart rate, respiratory rate and temperature; and low pH all predict increased mortality. Surgery within 1 week of ICU admission conferred a survival benefit. Finally, although severity of illness scores were worse in nonsurvivors than in survivors, no score reliably predicted mortality. The use of APACHE II score measured during the first 24 hours of ICU stay performs well as compared with the SHARF T0, SHARF II0 and Mehta scores, but it lacks perfect calibration.

## Key messages

• Severe AKI is responsible for about 10% of all ICU bed-days, and the presence of AKI doubles ICU and overall hospital lengths of stay.

• In adult, general critical care units in England, Wales and Northern Ireland, the mean age of patients with AKI in ICU is increasing and nonsurgical admissions now predominate, representing a change from the mid-1980s, when surgical conditions dominated.

• Although all severity of illness models tested have problems that lead to reduced performance, the UK APACHE II model when used in UK ICUs performs best.

• Traditional risk factors such as oliguria, CPR, mechanical ventilation, sepsis, length of stay more than 7 days before admission to ICU and extremes of physiology continue to be associated with the greatest increased risks for mortality.

## Abbreviations

AKI: acute kidney injury; APACHE: Acute Physiology and Chronic Health Evaluation; CMP: Case Mix Programme; CMPD: Case Mix Programme Database; CPR: cardiopulmonary resuscitation; ICNARC: Intensive Care National Audit & Research Centre; ICU: intensive care unit; RIFLE: Risk, Injury, Failure, Loss of kidney function, End-stage kidney disease; ROC: receiver operating characteristic; RRT: renal replacement therapy; SHARF: Stuivenberg Hospital Acute Renal Failure.

## Competing interests

The authors declare that they have no competing interests.

## Authors' contributions

PS, GL, AC and DH conceived of the study. DH was involved with data collection and statistical analysis. NK, PS and DH drafted the manuscript. All authors were involved in reviewing the manuscript.
